# Durability of Reinforced Concrete with Additions of Natural Pozzolans of Volcanic Origin

**DOI:** 10.3390/ma15238352

**Published:** 2022-11-24

**Authors:** Juan J. Santana, Natalia Rodríguez-Brito, Concepción Blanco-Peñalver, Vicente F. Mena, Ricardo M. Souto

**Affiliations:** 1 Department of Process Engineering, University of Las Palmas de Gran Canaria, 35017 Las Palmas de Gran Canaria, Spain; 2 Consejería de Obras Públicas y Transportes, Gobierno de Canarias, 38270 San Cristóbal de La Laguna, Spain; 3 Department of Chemistry, Universidad de La Laguna, P.O. Box 456, 38200 La Laguna, Spain; 4 Institute of Material Science and Nanotechnology, Universidad de La Laguna, 38200 La Laguna, Spain

**Keywords:** reinforced concrete, chloride penetration, carbonation, durability, useful life prediction, integral method, multi-regime method, modeling

## Abstract

In this work, the properties of concrete modified with dosages of natural pozzolans (NP) in substitution of cement or superfine aggregates were evaluated. Proportions of 20/80 pozzolan/cement or pozzolan/superfine aggregates were selected for the additions of quarry and tuff pozzolans. Pozzolanic activity, durability, compressive strength, characteristic resistance, settling consistency, density, electrical resistivity, depth of water penetration, accessible porosity, and carbonation and chloride penetration were determined for the resulting concrete mixtures, and they were subsequently compared to the values obtained for the reference concrete batches without additions. The results of the cementitious mixtures supplemented with tuff (PZT) and quarry (PZQ) pozzolans, expressed in mmol/L, are consistent with the pozzolanism test, with [Ca(OH)_2_]/[OH^−^] ratios at 7 days are 6.03/60.19 for PZQ and 1.78/92.78 PZT. In addition to the pozzolanic activity at these dosages, the characteristic resistance and durability parameters required by EHE-08 were verified. Particular attention was given to the determination of the diffusion of chloride ions, introducing an instrumental modification of the accelerated integral method. The modification provides values of diffusion coefficients similar to those obtained by the other methods with the advantage of greater stability and quality of the measurement.

## 1. Introduction

The reduction in CO_2_ emissions into the atmosphere has become a subject of great importance in recent decades in order to reduce the greenhouse effect [1]. Among the various types of industries, the cement sector is one of the largest contributors to CO_2_, estimated at around 8% of total anthropogenic greenhouse gas (GHG) emissions [2,3,4,5,6,7,8]. One of the ways to reduce this impact is to reduce cement consumption by partially replacing it with supplementary cementitious materials (SCMs), which can be by-products from industry or natural pozzolans (NP), such as fly ash (FA), ground granulated blast furnace slag (GGBS), silica fume (SF), metakaolin (MK), limestone (L), fine glass powder (GP), rice husk ash (RHA), and volcanic tuffs (natural pozzolans) [5,6,9,10,11,12,13,14,15,16,17,18,19,20]. These latter SCMs include both pozzolanic tuffs and those from the extraction of quarry aggregates. Indeed, the abundance of these materials in certain geographic areas, such as volcanic regions, makes them attractive candidates as a partial substitute for cement, mortar, and concrete [1,10,21,22,23,24,25]. These materials are often disposed of in landfills, thus constituting a major environmental problem [5]. Therefore, its reuse would reduce the environmental impact, apart from CO_2_ emissions, the reduction in the cost of concrete manufacturing, and the improvement of mechanical properties [17]. On the other hand, it is difficult to draw general conclusions on the behavior or to predict the reactivity of a given pozzolan, thus arousing a growing interest in the study of these tuffs or zeolitic deposits [26,27,28,29,30,31].

The effect of pozzolan additions to mortars and concretes varies depending on the type of pozzolan and the percentage of cement or aggregate substituted, among other factors. Generally speaking, pozzolan additions have been reported to increase durability [5,13,14,15,21,26,32,33,34,35,36,37,38], decrease the heat of hydration [1,38], increase resistance to sulfate attack [1,39], increase setting time, prevent water penetration [1,40], and reduce energy cost per unit of cement [37]. Specifically, the effect these additions have on mechanical strength depends on many factors, and conflicting results can often be found in the literature.

When natural pozzolans were used as a cement substitute in the range of 10–30%, Xia et al. [14] reported that the mechanical strength varied differently depending on the type of pozzolan, a fact corroborated by other authors [29,41]. Najimi et al. [1] found that the compressive strength was only affected slightly (actually less than 5%) by substituting about 25% of the Portland cement with natural tuff pozzolan. Costafreda et al. [11] recommend the industrial manufacturing of pozzolanic cement with the proportions 75:25% and 70:30% with natural pozzolans since they provide practically the same mechanical strength. Çullu et al. [42] observed that increasing the percentage of various natural pozzolans at the expense of cement produced a decrease in compressive strength, proposing that the optimum dose may be 10%. Turanli et al. [43] found that the compressive strength of mortar containing large amounts of volcanic ash was lower than that of Portland cement at any age less than 90 days. Khan et al. [18] found that improved fineness of volcanic ash up to 30% mass replacement of cement in mortar demonstrated compressive strength comparable to control and reference fly ash mortars at all ages. In recent work, these authors [19] demonstrated that the incorporation of those ultrafine volcanic ashes in percentages of 10% to 30% improved the compressive strength after 91 days of curing. These results, therefore, do not make it possible to extract a clear trend as to the effect exerted by the additions of natural pozzolans.

Regarding the penetration of aggressive substances, the addition of pozzolans leads to an improvement in the reduction in chloride ion penetration [1], but not in the carbonation process. Thus, Xia et al. [14] found that the addition of natural pozzolans caused a decrease in the resistance to carbonation and a decrease in the diffusion coefficients of chlorides, parameters related to the corrosion of reinforced concrete, chloride being the main cause of reinforcement corrosion [44,45]. The corrosive process has arisen interest in the last decades because it has been identified as the main process related to the durability of reinforced concrete, which leads to great economic losses and, on the other hand, a huge environmental cost due to increased CO_2_ emissions originated by the consumption of raw materials in the repair or replacement of damaged structures [46,47]. Although authors such as Kaid et al. [48] and Fajardo et al. [49] found that concrete mixed with natural pozzolan showed excellent corrosion resistance, quantification is necessary when these concretes are going to be used in areas with a high chloride content in the environment. For this reason, the penetration of aggressive agents is the basis for quantifying the durability of a structure, although there is no rigorous standardized methodology for predicting its useful life [50]. The first proposals for systematizing the notion of useful life were introduced in the 1970s [51,52] from the observation of the first degradations. These initiatives were by Tuutti reviewed these initiatives [53], and their use among researchers is currently widespread, even if their concretization in codes or recommendations is still very nascent [54].

In the present study, the behavior of concrete samples dosed with additions of natural pozzolans present in the Canary Islands (i.e., tuff pozzolan and quarry pozzolan) in substitution of cement or fine aggregates was tested by physico-chemical and mechanical assays according toto the regulations in force. The pozzolanic activity of the samples and the penetration of aggressive agents were evaluated and compared to samples without addition. The study of the penetration of chlorides in samples of reinforced concrete dosed with these additions stands out for its importance in the corrosion of the reinforcements, using the pool immersion method, the multi-regime test, and the integral method with an instrumental modification. In this way, the diffusion coefficients of the chlorides in the samples were determined. The durability of the tested mixtures was determined from the carbonation and chloride penetration models included in the Spanish regulation EHE-08 [54]. The feasibility of the additions was established based on the results obtained from mechanical and durability tests.

## 2. Materials and Methods

### 2.1. Materials and Sample Preparation

Two existing pozzolans in the study area, namely tuff (PZT) and quarry (PZQ) pozzolans, were investigated as possible additives for reinforced concrete. PZT comes from the crushing and grinding of rock classified as unwelded Ignimbrite lithotype (IGNS) [55]. They are made up of a chaotic mix of pumice fragments, ashes, rock fragments, and crystals. They have a trachytic or phonolitic composition, and petrographic microscopy reveals crystals of anorthoclase or sanidine, vitreous fragments of irregular morphology, fragments of aegirine, hornblende, and opaque minerals, all in a microcrystalline or vitreous matrix. PZT is a residue that comes from the clearing of an aggregate quarry located in the area (SATOCAN quarry in the municipality of Arico, Tenerife, Canary Islands). It is a powder with a real density or a specific weight of 2550 kg/m^3^, which is produced as waste during the exploitation in search of the optimal layers for the extraction of aggregates. Particle size analysis was performed for both pozzolans, and XFR analysis of PZT was also performed.

In this work, an II/A-P/42.5R MR-type cement was used, and Table 1 lists its chemical, physical and mechanical properties. Reinforced concretes were dosed in the laboratory with characteristic resistances 25, 30, and 35 N/mm^2^ (HA25, HA30, and HA 35, respectively) using this cement and additions of either PZT or PZQ. The concrete samples were assayed using the Fuller [56] method, and their main characteristics are given in Table 2. Pozzolans were introduced in a percentage of 20% by subtracting the amount of cement in the D3 samples, while in the D4 samples, the pozzolans were included as superfine aggregates by subtracting their weight from the basalt sand and not from cement. All proposed dosages and mixtures have been manufactured with expressed water/cement ratios (w/c), in which the moisture of the aggregates has been subtracted from the amount of water.

The pozzolanic activity index with lime (PAI) of the concretes with pozzolan contents was determined according to the ASTM C311/C311M-13 standard [57] and further established in the UNE 80303-2 standard [58] in item 7.2.b), and according to the test method UNE EN 196-5 [59] after 7 days.

The aggregates used were gravel and sand from a local quarry with CE marking. The basaltic aggregate, coded A, was selected from the typical quarry materials of the Canary Islands for the manufacture of concrete. These are gravel particles with an approximate size of 20 mm, chosen from the storage material of the assays.

The specimens of each sample were manufactured and stored in accordance with the UNE-EN 12390-1:2001 [60] and UNE-EN 12390-2:2009 [61] standards and according to the geometry established for each particular test. Thus, cylindrical specimens with a diameter of 15 cm and a height of 30 cm were fabricated for the tests of bending and compression, water penetration, chloride penetration by the pond method, direct resistivity, and Wenner. Next, cubic specimens with 10 cm edges were fabricated for chloride penetration tests using the integral method and accessible porosity. Sections 15 cm in diameter and 2.5 cm thick were employed in the multi-regime method. Finally, prismatic specimens of dimensions 15 cm × 15 cm × 60 cm were used in the natural and accelerated carbonation tests. For the integral method, a corrugated steel bar was embedded, such as armor, in each of the specimens. These bars were insulated and then laid out so that the exposed surface was 6 cm long, fully embedded in the concrete. In all cases, once the concrete was properly vibrated, it was hardened in a humid chamber for 28 days. After the hardening period, the specimens were subjected to various tests.

The samples were coded according to a letter-number-letter (*DXY*) sequence as described below, according to its definition according to EHE-08 [54], and taking into account their main characteristics, such as the grade and type of cement, the water/cement ratio and type of addition. The letter *D* indicates that the samples were mixed concrete made in the laboratory, whereas the digit *X* indicates the number of the mix, up to a total of 3 types of concrete dosages. They were followed by letters or acronyms, *Y*, which indicated the modifications that were made, depending on whether or not it included an addition (e.g., the letter *P*, to codify the concrete reference mixes, without addition; the letters PZT, for concrete mixes with the addition of pozzolan extracted from a block of tuff, and the letters PZQ, for concrete mixes with the addition of quarry pozzolan). Table 2 also shows sample coding and identification.

### 2.2. Tests and Methods

The concrete specimens were subjected to various tests as follows. Compressive strength at 28 days, according to UNE-EN 12390-3 [62], and resistance, according to UNE-EN 196-1:2005 [63]. Determination of fresh or settling consistency using the Abrams cone, according to UNE EN 12350-2 [64]. The density of hardened concrete UNE-EN 12390-7 [65]. Electrical resistivity using the direct method (Direct Resistivity) according to UNE 83988-1 [66] and by the four points or Wenner method (Indirect Resistivity) according to UNE 83988-2 [67]. Depth of penetration of water under pressure according to UNE 83309-90 [68] and UNE-EN 12390-8 [69]. Accessible porosity, according to UNE-EN 1936 [70] and according to UNE 83980:2014 [71]. Measurement of carbonation by the natural method at 90 days according to UNE 112011-94 [72], UNE 112011:2011 [73], and UNE 83993-1 [74], as well as by the accelerated method according to UNE 83993-2 [75]. Measurement of chloride penetration through the “Pond” arrangement according to PrUNE 83986 [76] and CEN/TS 12390-11 [77]. Determination of chloride content in hardened concrete according to UNE-EN 14629 [78]. The multi-regime test was performed according to UNE 83987 [79], whereas the integral method is described in UNE 83992-2 [80], and the polarization resistance was measured according to UNE 112072 [81]. Finally, the pozzolanic activity with lime (PAI) was determined according to ASTM C311/C311M-13 [57], UNE 80303-2 [58], and UNE EN 196-5 [59].

### 2.3. Modification of the Accelerated Integral Method

**Figure 1:**
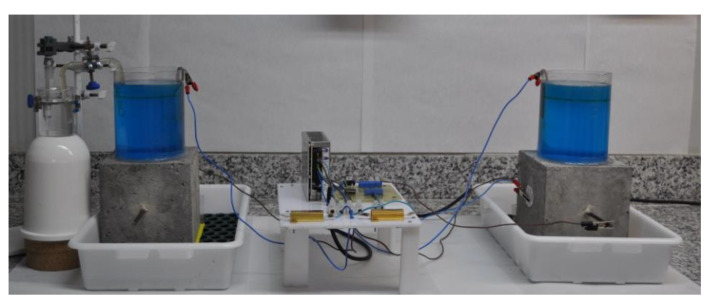
An assembly comprising two samples in parallel is used in the accelerated integral method.

The assembly of the experimental cell was carried out in accordance with the specifications in standard UNE 83992-2 [80]. Once the concrete specimen was prepared, PVC cylinders were attached to its top side to serve as containers for a 0.6 M NaCl + 0.4 M CuCl_2_ solution, and a Cu electrode was introduced to serve as the cathode of an electrochemical cell. The union of the plastic cylinder with the surface of the concrete specimen was sealed using neutral silicone, and the whole assembly is shown on the left side of Figure 1.

**Figure 2:**
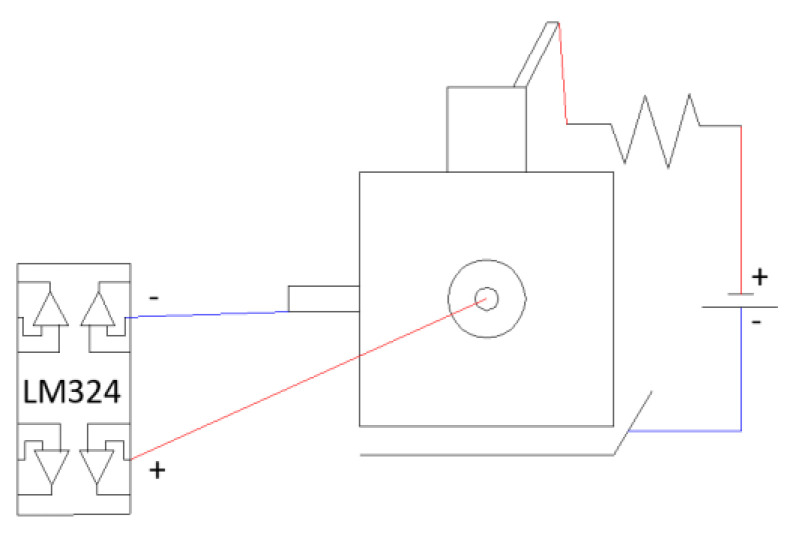
Wiring diagram of the implemented system. An electrode is fixed to the free side of the specimen on a plane perpendicular to that of the embedded bar.

Along with this setup, an instrumental modification is proposed here, which, although it does not affect the electrochemical nature of the assay, would improve the quality of the data for correct interpretation. Although the standard method indicates that a conventional reference electrode (calomel or Ag/AgCl, for instance) can be introduced into the attached electrochemical cell to determine the potential values of the embedded rods, we instead propose to include a fixed electrode along the plane perpendicular to the location of the embedded bar. This arrangement is shown schematically in Figure 2 and can be found for the specimen on the right of Figure 1.

While the standard method indicates a conventional reference electrode (either calomel or Ag/AgCl, etc.) can be introduced in the attached electrochemical cell in order to determine the potential values of the embedded bars, we propose instead to include a fixed electrode along the plane perpendicular to the location of the embedded bar. This arrangement is schematically described in Figure 2, and it can be observed for the specimen on the right in Figure 1. Thus, to measure a reference potential, the negative pole of the voltmeter is connected to the embedded steel, and the positive pole is to the electrode attached to the concrete. In this way, a measurement without fluctuations is obtained by having the constant reference of the external electrode. The two assemblies were set in parallel and with the same power supply potential in order to analyze any eventual differences arising. It was found that a voltage drop occurred after connecting the multimeter to the sample using the standard arrangement of the method. To overcome this problem, and taking into account that the values obtained at the time of connection and just before the voltage drop occurred were identical to those observed with the assembly proposed in the standard, an electrical circuit was included in the assembly, which would allow stabilization of the signal, although without affecting its value. The modification consists of introducing in the measuring clamp a control component powered by an external DC power supply, thus making it possible to measure data at any time without the drawbacks of the aforementioned instabilities.

**Figure 3:**
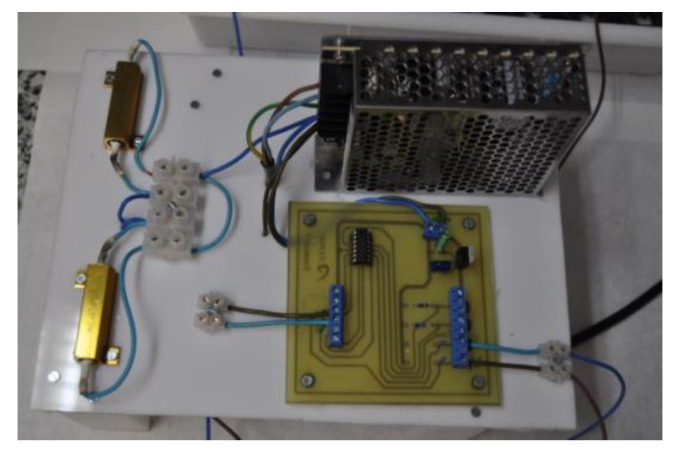
Voltage source and data acquisition system.

As can be seen in Figure 2 and Figure 3, the control element is an operational model LM324 which, powered by a TDK-Lambda power supply, model LS75-12, delivers a stable reading. The time drift and characteristic ripple of the power supply were characterized using a Pintek oscilloscope, model PS-605. A 0.1 Ω resistor in series with the potential source allowed measuring the current entering each probe without the need to disconnect the circuit. According to Ohm’s law, the intensity of the current circulating through a component of known resistance can be determined if we know the potential that is established across it.

### 2.4. Determination of the Durability

In order to assess the feasibility of the proposed dosages, the durability of the mixtures was determined using the carbonation and the chloride penetration models given in Annex 9 of EHE-08 [82]. Thus, the time required for carbonation at a distance *d* from the concrete surface was determined using the carbonation model:

(1) $$t={\left(\frac{d_{p}}{K_{c}}\right)}^{2}$$

where *d*_p_ is the carbonation depth in mm and *t* is the time in years. The carbonation factor *K_c_* was determined using:

(2) $$K_{c}=c_{\mathrm{env}}\cdot c_{\mathrm{air}}\cdot a\prime \cdot f_{\mathrm{cm}}^{b\prime }$$

where *f*_cm_ is the compressive strength of the concrete, in N/mm^2^, which can be estimated from the specified characteristic strength (*f*_ck_) as ($f_{\mathrm{cm}}=f_{\mathrm{ck}}+8$). *c*_env_ is the environmental factor, *c*_air_ is the air-entraining factor, and *a*’ and *b*’ are parameters which depend on the type of binder (see [82] for more details).

On the other hand, the time required for a certain chloride concentration *C*_th_ to occur at a distance *d* from the concrete surface is estimated according to the chloride penetration model by:

(3) $$t={\left(\frac{d_{p}}{K_{\mathrm{Cl}}}\right)}^{2}$$

where *d*_p_ is the depth in mm, and *t* is the time in years. The chloride penetration coefficient *K*_Cl_ is given by:

(4) $$K_{\mathrm{Cl}}=\alpha \sqrt{12D(t)}\left(1-\sqrt{\frac{C_{\mathrm{th}}-C_{b}}{C_{s}-C_{b}}}\right)$$

where *α* is a unit conversion factor equal to 56,157; *D(t)* is the chloride effective diffusion coefficient, for age *t*, expressed in cm^2^/s; *C*_th_ is the chloride concentration on the surface of the concrete, expressed in % of cement weight; *C*_b_ is the content of chloride from materials (aggregates, cement, water, etc.), when the concrete mix is prepared. The diffusion coefficient of chloride varies with the age of the concrete according to:

(5) $$D(t)=D(t_{0}){\left(\frac{t_{0}}{t}\right)}^{n}$$

where *D*(*t*_0_) is the chloride diffusion coefficient at age *t*_0_, *D(t)* is the coefficient at age *t*, and *n* is the age factor. The latter can be taken as equal to 0.5 in the absence of precise values determined by tests on the concrete considered. The value of *D(t*_0_*)* comes from EHE-08 [82], depending on the type of cement and the w/c ratio.

In order to predict the useful life in the design phase from the calculation of the initiation and propagation models described above, the following considerations were made for the starting data of certain parameters, which were kept fixed in all cases. The considered structure was classified as a tenement, whose minimum required value of the useful life in the project (*t*_g_) is 50 years. This implies a calculated useful life (*t*_d_) of 55 years according to Equation (6):

(6) $$t_{d}=\gamma_{t}\cdot t_{g}$$

The diameter of the steel bar (generic) was taken as Ø = 12 mm. Accordingly, on the basis of the EHE-08, the covers of the steel bar (*d*_r_) would be: (1) 20 mm for an IIa environment, with cement, additions, and a characteristic resistance between 25 and 40 N/mm^2^; (2) 25 mm for an IIb environment, with cement, additives and characteristic resistance between 25 and 40 N/mm^2^; or (3) 45 mm for an IIIa environment and our type of cement and projected useful life of 50 years. Regarding the environmental coefficient, the most unfavorable condition was selected, which corresponds to an “environment protected from the rain,” adopting the value *c*_env_ = 1. The aeration coefficient takes the value *c*_air_ = 1 for entrained air below 4.5%. The chloride content provided by the raw materials was set as *C*_b_ = 0. Although the critical concentration (*C*_th_, given as a percentage by weight of cement) should be 0.6% according to EHE-08, it was 0.4% for the samples tested using the pond method. Other parameters of the tested samples that are necessary for the calculation of the models (i.e., type of concrete, environment, water/cement ratio, and amount of cement) are introduced in the following Sections as employed.

The working life due to the corrosion of reinforcements was determined by adding together the initiation period and the corrosion propagation period, that in the case of corrosion by carbonation, is given by:

(7) $$t_{L}={\left(\frac{d_{p}}{K_{c}}\right)}^{2}+\frac{80}{\phi }\frac{d_{r}}{V_{\mathrm{corr}}}$$

In the case of corrosion by chlorides, this will be:

(8) $$t_{L}={\left(\frac{d_{p}}{K_{\mathrm{Cl}}}\right)}^{2}+\frac{80}{\phi }\frac{d_{r}}{V_{\mathrm{corr}}}$$

## 3. Results and Discussion

### 3.1. Characterization of the Components and the Concrete

**Figure 4:**
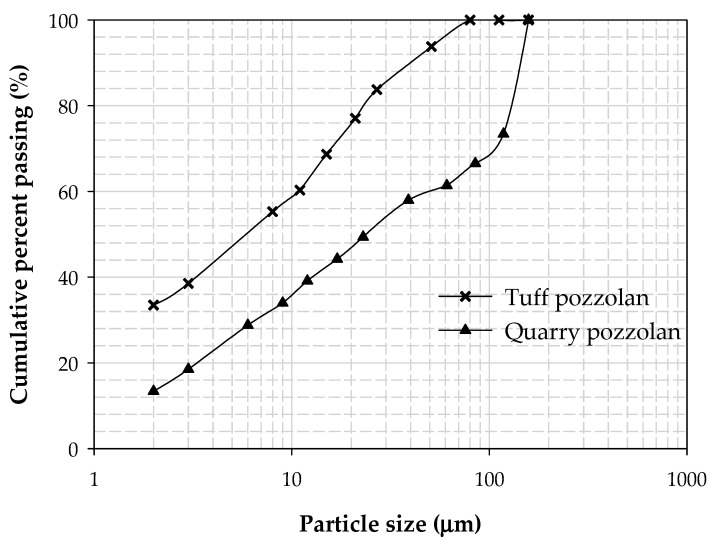
Particle size distribution of the used tuff and quarry pozzolans.

Table 3 shows the particle size distribution of the tuff and quarry pozzolans used as additives in this study, whereas Figure 4 shows their particle size distributions. In addition, the XRF analysis of the PZT is given in Table 4. Next, Table 5 summarizes the most relevant characteristics of the aggregates used.

Particle size distribution of the used tuff pozzolan and quarry pozzolan is shown in Figure 4. It can be seen that the tuff pozzolan had finer particles than the quarry pozzolan used in this study. The median particle size (50% passing size) of tuff pozzolan was almost 6 µm, while quarry pozzolan showed a particle size of 24 µm.

Table 6 presents the results of the flexural strength, compressive strength, and resistance activity index [58] of the additions with the reference cement. According to the test method of the UNE EN 196-1 standard [63], with a 75/25 mixture by weight, the compressive strength determined after 28 days must be equal to or greater than 75% of that corresponding to the standard cement I 42.5 R/S.

The reference samples, as well as the various additions, were characterized by means of the tests indicated in Section 2.3. Table 7 presents the results both from the tests carried out and from the calculation processes described in the standard that governs each of the tests.

**Figure 5:**
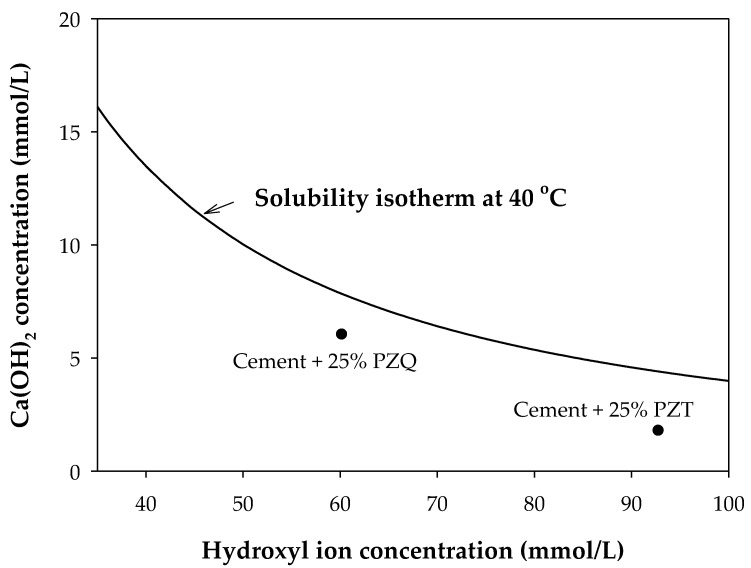
Variation of the pozzolanic behavior of the samples analyzed at 7 days.

The chemical analysis carried out for PZQ, according to ASTM C618 [83], shows a SiO_2_ + Al_2_O_3_ + Fe_2_O_3_ composition of 70.62% (type N pozzolan). This value is greater than the 70% set by the standard. Furthermore, the SO_3_ value is below 3%. On the other hand, the LOI value is greater than the 12% set by the standard. This abnormal value is justified by the late geochemical alteration of the sample due to corrosion, in particular of the pumice stone in the simple. In fact, analysis of samples taken near the quarry from which the material was obtained showed LOI values below 4% [47]. Both types of pozzolans showed pozzolanic activity according to the lime pozzolanic activity index (PAI). The results of the cement mixtures with the additions of PZT and PZQ, expressed in mmol/L, are consistent with the pozzolanism test (see Figure 5). The [Ca(OH)_2_]/[OH^−^] ratios at 7 days are 6.03/60.19 for PZQ and 1.78/92.78 PZT, with values under the saturation curve.

The additions of PZT and PZQ give concretes whose strengths correspond well to those required for concrete HA-25 (dosages D4) and HA-30 (dosages D3). When these are added to replace superfine aggregate, the pozzolan tuff shows increases of 22% and pozzolan quarry of 5% over the reference mix. When added as a replacement for cement, they cause a small decrease in resistance. All mixes exceed the required minimum characteristic strengths of 25 N/mm^2^ and 30 N/mm^2^, respectively. The dosages with the two pozzolans far exceed the limit of 75% of the resistant activity index with respect to the reference cement according to the UNE EN 196-1 standard. These results are in agreement with similar studies [1,47].

The additions cause a change in the consistency of the concrete, making it more fluid. Thus, according to the classification of the Abrams cone, it varies from plastic and liquid (between 3 and >16 cm) to the fluid (10 to 15 cm) and liquid (≥16 cm) range. The density values ranged between 2100 and 2500 kg/m^3^. In general, a decrease in the density is observed 24 h after demolding compared to fresh, followed by an increase in the density of the saturated specimens after 28 days of curing in a humid chamber, and finally, exhibits an expected decrease in the dry density, which varies between 3 and 10%. All of these increases and decreases in density are due to the increase or decrease in the amount of water in the sample.

The additions cause an appreciable change in the porosity of the samples. In the D3 dosages, with a lower water/cement ratio, the additions cause an increase in porosity that ranges from 31.23% in the case of tuff pozzolan to 18.71% in the case of quarry pozzolan. With regard to the D4 dosage, by maintaining the amount of cement and introducing the additions as superfine aggregate, the porosities were reduced in both cases by approximately 31%, thus improving the properties of the concrete. This variation in porosity complies with the results of water penetration required by EHE-08 for the durability control of concrete exposed in environments III and IV. In D3 concretes, water penetration results equal to or greater than the reference dosage are obtained, while for D4 dosages, in which the percentage of addition to the cement has not been subtracted, the results are not maintained but improved compared to the reference. The addition of pozzolan in the dosages with the highest water/cement ratio (i.e., 0.73%) effectively slows down the penetration of water.

It is also observed that the resistivity values obtained by the direct and the indirect (four-point) methods are close to 100 Ω m in all the batches. Pozzolanic additions cause a slight decrease in resistivity value when added to replace cement. However, when replacing the superfine aggregate, the tuff pozzolan causes a small increase in the resistivity values, while the quarry pozzolan causes almost no change in the measurement.

### 3.2. Modified Integral Method

As mentioned above, an instrumental modification was made to the integral method described in Section 2.3. From the measurement of the power supply ripple, a maximum of 15 mV AC was obtained at the 12 V DC output of the power supply. Note that, in the characteristics provided by the manufacturer, a maximum ripple of 120 mV is fixed for this model. On the other hand, we proceeded to obtain the drift over time of the source by performing two measurements at different times. The first was performed at time 0, which gave an output of 12.00 VDC. After 90 min, a second measurement was made, obtaining a value of 12.01 V on the screen, to obtain a value of 12.12 V after a month of testing.

**Figure 6:**
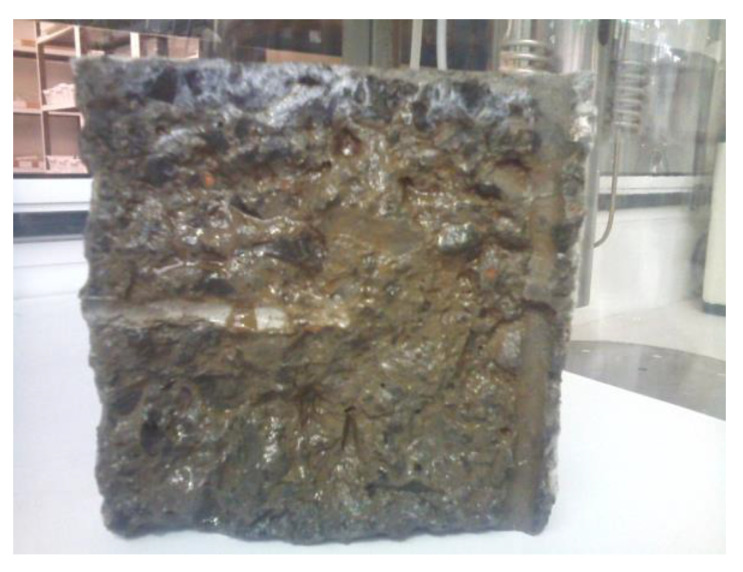
Chloride penetration profile revealed. The chloride penetration front can be seen in the upper part of the broken sample.

After a month of testing had passed, the concrete specimen used in the experimental device validation experiment was broken. A colorimetric technique was used to determine the chloride penetration. By adding a solution of silver nitrate (0.1 M) and the corresponding reaction of the compound with Cl^-^ ions, it was possible to see with the naked eye a whitish coloration when the two species came into contact. It must be noted that over time, this color turned purple until it almost disappeared 2 weeks after its development. As can be seen in Figure 6, the most unfavorable penetration of the chloride species reached a depth of 0.5 cm. Table 7 shows the values of the diffusion coefficients, as well as the time until the depassivation of the different specimens.

### 3.3. Penetration of Aggressive Species

Regarding the natural carbonation tests, among the results obtained, dosage 1 (D1P) stands out from the rest because it was not affected by the aggressive process, unlike the rest. In the case of the evolution of the carbonation process, by adding the pozzolans to the detriment of the cement, the natural process is hardly affected, but in the case of the accelerated tests, the carbonation fronts increase by 86.20% when adding tuff pozzolan and 106.79% in the case of quarry pozzolan (cf. Table 7). However, by subtracting superfine aggregate (dosages D4), the samples with additions show penetration rate values lower than the reference (D4P) both for the natural test (namely, −23.77 for D4PZT and −10.80 for D4PZQ) and for the accelerated test (−17.13 for D4PZT, and −6.77% for D4PZQ).

When evaluating the penetration of chlorides, it is found that the non-steady state diffusion coefficients are all of the same order of magnitude, regardless of the method with which they were obtained. The multi-regime method is the one that presents the lowest values of non-stationary diffusion coefficients compared to the other two methods. The reference sample D1P is the one that exhibits the lowest value of the non-stationary diffusivity coefficient. It is found that the additions of pozzolans to the detriment of the superfine aggregate lead to an improvement in the behavior of the dosages with respect to the penetration of chloride ions. When introduced in the part of cement, the dosages improve compared to the reference when analyzed with the multi-regime method but not when analyzed by the pond method, where mixtures containing quarry pozzolan show an increase in 9% in chloride penetration compared to the reference dosage, and of 5.5% in the case of tuff pozzolan. The chloride profiles follow a linear trend when calculated as a percentage of the sample weight with respect to the depth of penetration (mm), with regression indices R^2^ ranging from 0.891 (in the case of D2P) to 0.994 (for D4PZT).

According to the multi-regime method, mixes dosed with tuff pozzolan and quarry pozzolan have lower diffusion coefficients than their respective reference mixtures, whether they are introduced as either a cement or a superfine aggregate substitute. The greatest decreases in the coefficients were presented by the mixtures containing PZT, between 33 and 50%, and those containing PZQ, between 18 and 48%. These results coincide with those observed by other authors [17,84,85,86,87,88].

Finally, the modified integral method gives values similar to those obtained by the multi-regime method, with the advantage that this method provides information on the depassivation time of the reinforcement. A comparison with the reference dosages cannot be made as these have not been tested.

### 3.4. Estimation of Durability

Unfortunately, not all existing cement, according to RC-08 [89], are listed in the Durability Annex of the EHE-08 for the carbonation model. This was the case with the cement II/A-P/45.5 R/MR used in the tested samples. For this reason, this cement had to be considered in the model as Portland cement binder or Portland cement + 28% fly ash. On the other hand, an experimental comparison was made to observe the difference between using *f*_ck_ + 8 as the characteristic strength or, alternatively, the average compressive strength of cement, *f*_cm_. Table 8 shows the results with the raw data provided by the EHE-08, whereas Table 9 shows the results obtained from the tests carried out. For the resistance data variable, the average compressive strength *f*_cm_ of each set was taken, and three options were considered to catalog the type of binder, namely, Portland Cement, Portland Cement + 28% fly ash, and Portland Cement + 9% silica fume.

To estimate the durability by applying the chloride penetration model (Equations (3) and (4)) and the data provided by EHE-08, the diffusion coefficient, the surface, and the critical chloride concentrations were introduced, together with the chloride penetration data obtained by means of the pond method. Table 10 shows the results obtained with the data provided by the EHE-08 when considering *D*(*t*_0_) for a CEM II/A-V cement according to its water/cement ratio. Therefore, the value of *D*(*t*) is determined for *t* = 50 years (Equation (5)), while *t*_0_ is taken at 28 days (i.e., 0.0767 years). For the calculation of *K*_CL_, the parameter *C*_th_ by weight of cement is 0.6%, whereas the concentration of chlorides on the surface in weight of cement, *C*_s_, was taken in all cases as the value corresponding to an IIIa environment (up to 500 m from the sea the shore) of 0.14%, although in reality only the reference sample D1 was manufactured for the IIIa environment. The lifetime values (*t*_L_) thus determined were of the same order as those obtained using the carbonation model. All three dosages were found to be well above the design value (*t*_d_) set at 55 years, confirming that the reference doses are adequate for use in real-world applications. It also predicts that dose D3 (HA-30/F/20/IIb) is the most durable according to the model, followed by D1 (HA-35/P/20/IIIa), although it should be noted that the *V*_corr_ of the latter is always higher due to the assumption of operation in an IIIa environment.

Table 11 presents the data for the calculation of the useful life of all the mixed samples, with the data provided by the EHE-08 and the value of *D*(*t*_0_) taken from each of the samples tested for chloride penetration from the pond method. For the derivation of *D*(*t*), *t* was set as 50 years, and *t*_0_ as 90 days (0.2466 years); and for the calculation of the chloride penetration coefficient (*K*_CL_), the critical concentration (*C*_th_) by weight of cement was 0.4%, and the concentration of chlorides at the surface by the weight of cement (*C*_s_) corresponded to that of each sample.

By applying the durability model for corrosion processes considering carbonation as the relevant process determining the initiation period and using the data provided in the EHE-08, it is observed that the results greatly exceed the design value (*t*_d_) established in 55 years (cf. Table 8). The three dosage types are suitable for use in fulfilling the conditions of the proposed construction element, being the dosage D3 (HA-30/20/F/IIb), the most durable according to the model, followed by D1 (HA-35/20/P /IIIa). This fact has been observed by other authors [13,33,35].

When introducing the test results of the dosages in the carbonation model (see Table 9), it is observed that the additions of tuff and quarry pozzolans cause an increase in the useful lifetime (*t*_L_) which greatly exceeds the design value (*t*_d_), of 55 years, as well as the value obtained for the reference mixes. Regarding the approach of cataloging the binder in the different batches, it seems that the choice of Portland cement +28% ash in the modeling is the most conservative, although it gives higher values than those of the useful life calculation.

When applying the chloride penetration model for the estimation of durability, the estimated useful life (*t*_L_) values for the reference batches greatly exceed the design value (*t*_d_) set in 55 years, which confirms that the reference dosages are suitable for the use and conditions of the proposed building element. Here again, dosage D3 (HA-30/F/20/IIb) appears to be the most durable according to the model, followed by D1 (HA-35/P/20/IIIa), although it should be clarified that the *V*_corr_ of the latter is always superior due to the premise of being an IIIa environment.

During the introduction of the pozzolanic additions, we observe that for the case of the D1P sample, the results obtained in the prediction from the experimental data largely exceed the estimate made by the EHE-08, while the additions decrease the service life considerably, although all exceed the design value of the useful life (i.e., *t*_d_ = 55 years).

## 4. Conclusions

All laboratory concrete mixes, both reference and added with tuff or quarry pozzolans, whether used as a substitute for cement or for the superfine aggregate in a percentage of 20%, exceed the minimum characteristic resistance and the durability values required by EHE-08 on the basis of the carbonation and chloride penetration models. Both types of pozzolans showed pozzolanic activity according to the lime pozzolanic activity index (PAI) with [Ca(OH)_2_]/[OH^−^] ratios at 7 days are 6.03/60.19 for PZQ, and 1.78/92.78 for PZT.

In general, when replacing the cement, the additions cause an increase in porosity, a decrease in resistivity, and an increase in the depth of the carbonation front, while when replacing the superfine aggregates, they cause a decrease in porosity and a decrease in the carbonation front. The added mixes change their consistency with respect to the reference, so the introduction of the pozzolans influences the consistency of the mixes, making them more fluid.

The non-stationary diffusion coefficients obtained by the three methods are of the same order of magnitude, the highest values being those obtained by the pond method, followed by the integral method, and finally by the multi-regime method. In the pond immersion test, when the pozzolans replaced the cement, an increase in the penetration of chlorides compared to the reference dosage was observed. In the rest of the cases, a lesser diffusion of chlorides was observed when pozzolans replaced cement or superfine aggregates. There were decreases between 33 and 50% for the diffusion coefficients in the cementitious mixtures containing PZT, while those containing PZQ showed a more moderate decrease which varied between 18 and 48%.

## Figures and Tables

**Table 1:** Chemical, physical, and mechanical properties of the cement (type II/A-P/42.5R MR).

	II/A-P/42.5R MR
Loss on ignition (LOI)	3.4
Insoluble residue	10.1
Chloride (Cl^−^, %)	0.01
Sulphates (SO_3_, %)	3.38
Initial setting time (min)	150
Pozzolan content (%)	19
Clinker content (%)	76
Gypsum content (%)	5
End of hardening (EH, min)	215
Compressive strength at 7 days (N/mm^2^)	37.0
Compressive strength at 28 days (N/mm^2^)	51.6

**Table 2:** Identification, classification and characteristics of all concrete samples tested. All were made with type II/A-P/42.5R MR cement.

Code	D1P	D3P	D3PZT	D3PZQ	D4P	D4PZT	D4PZQ
Type of Aggregates	Reference	Reference	Tuff Pozzolan	Quarry Pozzolan	Reference	Tuff Pozzolan	Quarry Pozzolan
Characteristic resistance (N/mm^2^)	HA-35	HA-30	HA-30	HA-30	HA-25	HA-25	HA-25
Cement content (kg/m^3^)	325	300	240	240	300	300	300
Pozzolan content (kg/m^3^)	-	-	60	60	-	60	60
Basalt gravel (kg/m^3^)	806.19	945.75	945.75	945.75	945.75	945.75	945.75
Basalt sand (kg/m^3^)	688.08	502	502	502	502	442	442
African sand (kg/m^3^)	250.23	296.75	296.75	296.75	296.75	296.75	296.75
Water content (L/m^3^)	156	167	167	167	198	195	195
Cement ratio (%)	0.51	0.56	0.57	0.57	0.76	0.73	0.73

**Table 3:** Particle size analysis of additives by wet sieving.

	Tuff Pozzolan	Quarry Pozzolan
UNE Sieves	% Passing Through	
0.158	16.02	16.02
0.112	14.38	14.38
0.080	12.75	12.75
0.051	11.11	11.11
0.027	7.85	7.85
0.021	6.21	6.21
0.015	6.21	6.21
0.011	6.21	6.21
0.008	4.58	4.58
0.003	2.94	2.94

**Table 4:** XRF analysis of the tuff pozzolan.

SiO_2_	Al_2_O_3_	Fe_2_O_3_	CaO	MgO	Na_2_O	K_2_O	MnO	P_2_O_5_	TiO_2_	LOI	SO_4_
50.19	17.35	3.08	1.45	1.31	6.42	4.94	0.15	0.10	0.64	14.38	0.21

**Table 5:** Origin, size, and absorption coefficient of the aggregates.

Aggregate	Origin	Size	Absorption Coefficient
Sand	Siliceous	0/2	1.50
Sand	Basaltic	0/4	3.95
Gravel	Basaltic	10/20	3.65

**Table 6:** Flexural strength, compressive strength, and resistance activity index of the additives in the reference cement.

Sample	Aggregate	Flexural Strength (N/mm^2^)	Compressive Strength (N/mm^2^)	RAI (%)
Cement (I/42.5 R/SR)	Reference	10.1	5.4	100
75% Cement + 25% PZT	Tuff pozzolan	9.1	5.4	100
75% Cement + 25% PZQ	Quarry pozzolan	8.7	4.8	88.9

**Table 7:** Experimental results of the tests made on the concrete mixtures.

Code	D1P	D3P	D3PZT	D3PZQ	D4P	D4PZT	D4PZQ
Type of aggregates	53	52.5	54.7	54.7	52.5	54.7	54.7
Consistency (cm)	4	15	15	19	10	11.5	10.5
Fresh density (kg/m^3^)	2399	2475	2379	2423	2335	2365	2365
Density after 24 h (kg/m^3^)	2421	2449	2356	2408	2320	2343	2353
Density after 28 d (kg/m^3^)	2428	2468	2391	2437	2339	2364	2374
Dry density (kg/m^3^)	2324	2359	2255	2282	2110	2269	2278
Accumulated porosity (%)	14.33	15.5	18.4	20.34	22.5	15.59	15.4
RCS of concrete (N/mm^2^)	57	52.79	51.34	50	38.14	46.54	43.72
RIM after 28 d (Ω m)	115.4	109.9	106.6	79.4	107.5	120	105.3
RIC after 28 d (Ω m)	109.5	111.9	104.1	80.7	99.8	120.6	95.3
RID after 28 d (Ω m)	112	121.8	109.8	86.8	104.8	118.3	103.1
Natural *V*_CO2_ (mm/year^½^)	0	1.41	1.41	1.54	3.15	2.4	2.81
Accelerated *V*_CO2_ (mm/year^½^)	0	7.55	14.05	15.6	17.67	14.64	16.48
Profile *X*_Cl_ (mm)	4.2	11.8	13.5	13	13.8	13	14.8
Profile *V*_Cl_ (mm/year^½^)	8.46	23.66	27.04	26.04	27.85	26.18	29.7
Pond, *D*_ns_ × 10^−8^ (cm^2^/s)	0.6329	4.1	6.363	7.789	7.205	4.492	5.841
Multi-regime, *D*_ns_ × 10^−8^ (cm^2^/s)	3.0516	3.5094	2.3337	2.8636	4.8058	2.3694	2.4799
Initial/final intensity (mA)			5.2/9.45	5.72/15.71		4.89/8.33	5.62/10.17
Initial/final resistance (Ω)			2.307/1.270	2.098/0.764		2.045/1.440	2.135/1.180
Integral, *D*_ns_ × 10^−8^ (cm^2^/s)			4.019	3.191		4.019	6.028
Depassivation time (s)			2,332,800	2,937,600		2,332,800	1,555,200

**Table 8:** Application of the carbonation model for the general calculation method for the useful life. Carbonation model according to EHE-08 data.

Sample	*f*_ck_ + 8 (N/mm^2^)	*K*_c_	*t*_i_ (Year)	*d*_r_ (mm)	*V*_corr_ (μm/Year)	*t*_p_ (Year)	*t*_L_ (Year) (*t*_L_ = *t*_i_ + *t*_p_)
*f*_ck_ + 8, and binder coefficients, *a*′ = 360 and *b*′ = −1.2							
D1	43	3.95	57.80	45	20	15	72.80
D3	38	4.58	42.97	25	2	83.33	126.30
D4	33	5.42	30.62	20	3	44.44	75.07
D1	43	3.95	57.80	45	20	15	72.80
*f*_ck_ + 8, and binder coefficients, *a*′ = 1800 and *b*′ = −1.7							
D1	43	3.01	99.42	45	20	15	114.42
D3	38	3.71	65.31	25	2	83.33	148.64
D4	33	4.72	40.42	20	3	44.44	84.87

**Table 9:** Application of the carbonation model for the general calculation method of the useful life. These values were obtained for each type of conglomerate using data extracted from the batches, and the average resistance of the concretes (*f*_cm_).

Sample	*f*_cm_ (N/mm^2^)	*K*_c_	*t*_i_ (Year)	*d*_r_ (mm)	*V*_corr_ (μm/Year)	*t*_p_ (Years)	*t*_L_ (Year) (*t*_L_ = *t*_i_ + *t*_p_)
Type of binder, Portland cement (*a*′ = 1800 and *b*′ = −1.7)							
D1P	57.00	1.86	259.22	45	20	15	274.22
D3P	52.79	2.12	199.70	25	2	83.33	283.03
D3PZT	51.34	2.23	181.66	25	2	83.33	264.99
D3PZQ	50.00	2.33	166.03	25	2	83.33	249.37
D4P	38.14	3.69	66.13	20	3	44.44	110.57
D4PZT	46.54	2.63	130.11	20	3	44.44	174.55
D4PZQ	43.72	2.92	105.20	20	3	44.44	149.64
Type of binder, Portland cement + 28% fly ash (*a*′ =360 and *b*′ = −1.2)							
D1P	57	2.81	113.69	45	20	15	128.69
D3P	52.79	3.08	94.57	25	2	83.33	177.91
D3PZT	51.34	3.19	88.46	25	2	83.33	171.79
D3PZQ	50	3.29	83.02	25	2	83.33	166.35
D4P	38.14	4.56	43.35	20	3	44.44	87.79
D4PZT	46.54	3.59	69.89	20	3	44.44	114.34
D4PZQ	43.72	3.87	60.15	20	3	44.44	104.60

**Table 10:** Application of the chloride penetration model for the general calculation method of the useful life according to data included in the EHE-08.

Sample	*D*(*t*_0_) × 10^−7^ (cm^2^/s)	*D*(*t* = 50) × 10^−9^ (cm^2^/s)	*K*_CL_ (mm/Year^½^)	*t*_i_ (Year)	*d*_r_ (mm)	*V*_corr_ (μm/Year)	*t*_p_ (Year)	*t*_L_ (Year) (*t*_L_ = *t*_i_ + *t*_p_)
D1P	0.90	3.53	2.56	137.14	45	20	15.00	152.14
D3P	1.09	4.27	3.21	87.49	25	2	83.33	170.83
D4P	1.49	5.84	3.75	64.00	20	3	44.44	108.45

**Table 11:** Application of the chloride penetration model for the general calculation method of the useful life using data obtained from the pond immersion test.

Sample	*D*(*t*_0_) × 10^−8^ (cm^2^/s)	*D*(*t* = 50) × 10^−9^ (cm^2^/s)	*K*_CL_ (mm/Year^½^)	*t*_i_ (Year)	*d*_r_ (mm)	*V*_corr_ (μm/Year)	*t*_p_ (Year)	*t*_L_ (Year) (*t*_L_ = *t*_i_ + *t*_p_)
D1P	0.63	0.45	2.15	194.47	45	20	15.00	209.47
D3P	4.10	2.88	6.35	22.32	25	2	83.33	105.66
D3PZT	6.36	4.47	7.49	16.06	25	2	83.33	99.39
D3PZQ	4.88	3.43	6.86	19.13	25	2	83.33	102.46
D4P	7.21	5.06	8.78	11.68	20	3	44.44	56.12
D4PZT	4.49	3.15	6.82	19.34	20	3	44.44	63.79
D4PZQ	5.84	4.10	7.72	15.12	20	3	44.44	59.56

## Data Availability

The raw/processed data required to reproduce these findings cannot be shared at this time as the data also forms part of an ongoing study.
